# Endophytic fungi: hidden treasure chest of antimicrobial metabolites interrelationship of endophytes and metabolites

**DOI:** 10.3389/fmicb.2023.1227830

**Published:** 2023-07-11

**Authors:** Priyanka Jha, Tamanna Kaur, Ishita Chhabra, Avirup Panja, Sushreeta Paul, Vijay Kumar, Tabarak Malik

**Affiliations:** ^1^Department of Biotechnology, Lovely Faculty of Technology and Sciences, Lovely Professional University, Phagwara, Punjab, India; ^2^Metropolis Healthcare Ltd., New Delhi, India; ^3^Amity Institute of Biotechnology, Amity University, Kolkata, West Bengal, India; ^4^Biomedical Sciences, Institute of Health, Jimma University, Jimma, Ethiopia

**Keywords:** secondary metabolites, terpenoids, symbiosis, alkaloids, endophytic fungi

## Abstract

Endophytic fungi comprise host-associated fungal communities which thrive within the tissues of host plants and produce a diverse range of secondary metabolites with various bioactive attributes. The metabolites such as phenols, polyketides, saponins, alkaloids help to mitigate biotic and abiotic stresses, fight against pathogen attacks and enhance the plant immune system. We present an overview of the association of endophytic fungal communities with a plant host and discuss molecular mechanisms induced during their symbiotic interaction. The overview focuses on the secondary metabolites (especially those of terpenoid nature) secreted by endophytic fungi and their respective function. The recent advancement in multi-omics approaches paved the way for identification of these metabolites and their characterization via comparative analysis of extensive omics datasets. This study also elaborates on the role of diverse endophytic fungi associated with key agricultural crops and hence important for sustainability of agriculture.

## 1. Introduction

Numerous microorganisms exist in mutualistic associations with plants, whereby their interaction results in a beneficial outcome for both entities. Microbes with such capabilities are ample in above-ground as well as in underground (edaphic) habitats. Epiphytes are microorganisms that are found on the external surface of plants, whereas endophytes are microorganisms that reside and establish themselves within plant tissues, specifically within the leaves or roots. Among these, the rhizosphere harbors a plethora of active microorganisms that enhance plant nutrition, growth, and development of plants. The rhizosphere can be defined as the soil zone in the proximity of plant roots that is affected by the presence of microorganisms and root exudates, including compounds secreted by the root system of the plants (Kanchan et al., 2020). The region is characterized by multifaceted plant-microbe interactions, encompassing mutualistic, symbiotic, or parasitic relationships. These interactions are capable of triggering the biosynthesis of secondary metabolites by the microbial cells, which can be advantageous for the plants by conferring tolerance to biotic and abiotic stresses contributing to the plant’s overall fitness (Koza et al., 2022). Endosymbiotic microorganisms, which are typically bacteria and/or fungi, establish mutually beneficial relationships with their plant host including improved host fitness. These organisms are considered to be non-pathogenic since they can complete a part of their life cycle or even the whole life cycle within their host without causing any symptoms of disease (Nair and Padmavathy, 2014). The endophytic community can be further divided into two subgroups which involve ‘obligate endophytes’ (endophytes that critically dependent on plant metabolism for their survival) and ‘facultative endophytes’ (endophytes that spend certain stages of their life cycle outside the host body and are mostly associated with plants present in their nearby soil environment) (Hardoim et al., 2008; Abreu-Tarazi et al., 2010).

Of all the microorganisms inhabiting the rhizosphere, endophytic fungi or fungal endophytes have garnered significant attention from researchers. This is owing to their ability to produce various bioactive molecules such as antibacterial compounds, and biostimulants, which can facilitate essential oil biosynthesis (Radic and Strukelj, 2012; Uzma et al., 2018; El Enshasy et al., 2019). Essential oils are the aromatic compounds that have high vapor pressure and low boiling point. Vanillin and 3-methoxy-4-hydroxytoluene was isolated from the roots of *Zingiber officinale* Rosc. infected with *Streptomyces aureofaciens* SMUAc130 (Taechowisan et al., 2005). The bacterial volatile compounds are significant in establishing interspecific and or intraspecific communication and role in defence mechanism against antagonistic microbes. Volatile organic compounds (VOCs) play extremely important role in antibiosis and signaling mechanism for microbes with symbiotic relation under competitive soil conditions (Polito et al., 2022). Endophytic fungi have been recognized for their multifaceted roles in plant-microbe interactions: nutrient solubilization in the plant rhizosphere, promoting plant growth, acting as biocontrol agents against pests and pathogens, inducing systemic resistance against biotic and abiotic stresses, and participating in the biosynthesis of secondary metabolites (Mehta et al., 2019; Poveda et al., 2020a; Cui et al., 2021; Poveda et al., 2021; Poveda and Baptista, 2021). Fungal secondary metabolites may act by causing alterations in the host plant’s morphology and physiology (Alam et al., 2021). Endophytic fungi establish symbiotic associations with host plants through a process that involves the enzymatic degradation of the host plant’s cell wall. To colonize the host plant tissue, endophytic fungi produce a range of cell wall-degrading enzymes including cellulase, laccase, pectinase, and xylanase. These enzymes facilitate the infiltration, colonization, and proliferation of endophytic fungi within the host plant tissue by altering the physical and structural properties of the host cell wall (Chow and Ting, 2019). Upon degradation of the host plant cell wall, host plant activates its defence mechanisms such as pattern-recognition receptors (PRRs) which provide innate immunity to the plant. In order to survive in plants, the endophytes require specific strategies to avoid being detected by the plant based immune system. One such strategies include, hiding of chitin by modifying it or oligomers derived from chitin employing chitin deacetylases (Cord-Landwehr et al., 2016). Apart from these, many fungal endophytes have been identified to secrete bioactive compounds known as secondary metabolites, which are helpful in coping with biotic and abiotic stresses. These secondary metabolites include mostly alkaloids, terpenoids, polyketides. Besides these major ones, certain secondary metabolites are also secreted such as flavonoids, saponins, phenols, and phenolic acids, aliphatic and chlorinated metabolites, peptides, and steroids. It was found that similar secondary metabolites are being produced by the fungal endophytes and even in larger concentrations as compared to the plants. Using gene clustering, transcription factors and horizontal gene transfer, we can study the biosynthesis pathways and genes encoding specific types of bioactive molecules.

Fungal endophytes are emerging source for terpenoids, an important class of secondary metabolites. Terpenoids are a diverse group of natural products, which consist of C5 isoprene units and are the largest class of natural products (Perveen, 2018). The structural diversity of terpenoids is substantial, with more than 80,000 terpenoids having been identified from both plant and microbial sources. The isoprene units required for terpenoid biosynthesis are typically synthesized through either the mevalonic acid pathway (MVA) or the methylerythritol phosphate pathway (MEP). Terpenoids exhibit a diverse range of pharmacological and nutritional activities, as well as utility in the food and cosmetic industries. Moreover, their therapeutic potential has been explored in the context of COVID-19 treatment due to their notable antiviral properties (Diniz et al., 2021). Due to these activities, there is increase in demand of terpenoids which imposes a strain on the terpenoid-producing plant species. A wide variety of terpenoids are produced by endophytic fungi but none of them is capable of producing terpenoids at commercial scale as their yield is low, especially after repeated subculturing.

Initially, with the advent of first generation sequencers alongwith amplicon based sequencing for variable regions of genomes was being employed for deciphering microbiome understanding. However, extensive studies on root morphology and root exudation in deciphering rhizobiomes can be done employing next generation sequencing (NGS). Metagenomics allows detailing of complete genomic information by assembling DNA sequences into genes. It also provides information about novel genes, bio based-products, biomolecules, interaction between microbial communities. Whereas, transcriptomics utilizes the NGS based information to identify and quantify the presence of the particular RNA molecule in biological samples. Metabolomics, on the other hand is a technique which is utilized to identify and analyze metabolite changes occurring due to overexpression or mutation in a desired gene (Chen et al., 2022). The combination of various omics-based approach can help in better understanding of plant-endophyte interaction mechanisms. This study focusses on the secondary metabolites (especially those of terpenoid nature) secreted by endophytic fungi. The study elaborates the role of endophytic fungi as potential source of terpenoid producer alongwith its host related molecular interactions. Concomitantly, a noteworthy discussion on recent multi-omics based approaches employed to have better understanding of host and endophytic fungi interaction for secondary metabolite production.

## 2. Endophytes: fungal association with plants

Endophytic fungi (EF), also known as fungal endophytes, are communities of fungi that form associations either inter- or intracellularly with host plant tissues while simultaneously providing benefits to the host and gaining benefits from it (Alam et al., 2021). These fungi establish a mutualistic or symbiotic relationship with the host plant, and are categorized into two groups based on this relationship. The first group is the Clavicipitaceous fungi or Balansiaceous group, which includes class I endophytes. This group comprises free-living symbiotic species that survive in cool season grasses (Poaceae), infect the ovules of host plants, and are transmitted vertically to progenies through host seeds. The living rhizomes and shoots of the host plant are colonized by these endophytes. The second group is the Non-Clavicipitaceous fungi or Non-Balansiaceous group, which includes class II, class III, and class IV endophytes. The Class I endophyte species are considered as obligate endophytes that offer protection to their host plant under conditions of drought stress, by secreting bioactive metabolites that exhibit defensive or supportive properties (Roberts and Lindow, 2014; Poveda et al., 2021). Non-Clavicipitaceous fungi or Non-Balansiaceous group (includes class II, class III and class IV endophytes) are non-grass host related groups having extensive biodiversity and distribution. These endophytes reside within plant tissues in a dormant or quiescent state without causing any apparent harm to the host plant, that is, they are not closely associated with the host plant. However, as soon as the chemical changes in the host plant occurs either through injuries or environmental stresses, these EFs then enter the host plant intracellularly through roots shoots or rhizomes (Carroll, 1988; Rodriguez et al., 2008; Mishra Y. et al., 2021). In Class II endophytes colonization of the host tissue occurs through roots, shoots and rhizomes whereas colonization occurs through shoots only and roots only in class III and class IV endophytes, respectively. The transmission occurs both vertically and horizontally in class II endophytes whereas only horizontally in class III and class IV endophytes (Rodriguez et al., 2009). Brief list of important crops and beneficial endophytic fungi with their respective functions have been provided in Table 1.

**Table 1:** Important crops and beneficial endophytic fungi with their respective functions.

Agricultural Crop	Beneficial Endophytic Fungi	Function	Reference
Rice	*Phoma glomerata, Penicillium simplicissimum, Galactomyces geotrichum, Fusarium oxysporum, Phoma sp., Aspergillus ustus*	Growth promoting factors, Salt tolerance, improves mineral nutrition and quality especially, under low nitrogen content in soil, ameliorates crop production.	Tang et al. (2022) and Potshangbam et al. (2017)
Wheat	*G. etunicatum, G. intraradices, G. fasciculatum T. atroviride, Glomus spp., Trichoderma atroviride, Alternaria alternata*	Improves germination of seed, rate of the shoot and root growth, chlorophyll content, alleviates drought tolerance and ameliorates crop yield.	Qiang et al. (2019), Saxena et al. (2013), and Colla et al. (2015)
Maize	*Gibberella fujikuroi, Fusarium oxysporum, Fusarium sacchari, Gibberella intermedia, Trichoderma atroviride, Aspergillus awamori, Metarhizium robertsii, Sarocladium zeae*	Plant growth promoting factors, induces disease resistance by improving plant immune response, protects against black cutworm larvae, improves crop yield	Ahmad et al. (2020), Mehmood et al. (2019), Contreras-Cornejo et al. (2018), Potshangbam et al. (2017), and Renuka and Ramanujam (2016)
Citrus	*Alternaria alternata, Alternaria citri, Alternaria rosae, Alternaria sp., Aspergillus sp., Colleotrichum karstii, Diaporthe eres, Piriformospora indica, Cladosporium sp., Pseudozyma sp., Meyerozyma sp.*	Acts as a biocontrol agent against pathogenic bacteria and fungal species, improved soil quality by enhancing the total phosphatase activity, and better fruit quality due to enhanced macronutrient content.	Cheng et al. (2022), Nicoletti (2019), and Sadeghi et al. (2019)
Banana	*Fusarium sp., Phoma sp., Nigrospora sp., Penicillium sp., Colleotichum sp., Piriformospora indica*	Tolerance to cold stress, resistance to pathogenic fungi, protects from nematode infection, increases productivity.	Kisaakye et al. (2023), Li et al. (2021), and Zakaria and Aziz (2018)
Soybean	*Penicillium minioluteum, Porostereum spadiceum, Rhizopus oryzae, Paecilomyces formosus*	Protects from abiotic salinity and thermal stress, helps in promoting photosynthetic activity, improves plant growth by increased macronutrient uptake, disease resistance.	Bilal et al. (2020), Ismail et al. (2020), Bajaj et al. (2018), Hamayun et al. (2017), and Khan et al. (2012)
Tomato	*G. intraradices, T. atroviride, Fusarium solani, Pochinia sp., Pythium sp., Piriformospora sp.*	Acts as biofertilizers by improving the nitrogen content of the soil, Increases biomass production, counteracts bacterial pathogen attacking the plant	Sinno et al. (2020), Pappas et al. (2018), and Colla et al. (2015)
Potato	*Rhizophagus irregularis, Epicoccum nigrum, Curvularia lunata*	Mitigates oxidative stress in roots and shoots during plant growth, acts as a biocontrol agent against blackleg disease of potato and has antimicrobial properties.	Deja-Sikora et al. (2020), Bagy et al. (2019), and Avinash et al. (2015)
Sunflower	*Penicillium citrinum, Talaromyces assiutensis, Aspergillus terreus, Rhizophus oryzae, Brassica napus, Piriformospora indica*	Alleviates thermal stress, disease resistance, act as biocontrol agent against pathogenic fungi, mitigates cadmium toxicity and helps in improving chlorophyll content under stress	Farhat et al. (2023), Ismail et al. (2020), Shahabivand et al. (2017), and Waqas et al. (2015)
Cotton	*Penicillium simplicissimum, Leptosphaeria sp., Talaromyces flavus, Acremonium sp., Beauveria bassiana, Purpureocillium lilacinum*	Protects against cotton wilt against Verticillium sp., promotes plant growth, protects the host plant against aphid and control crop infestation.	Yuan et al. (2017) and Lopez and Sword (2015)

### 2.1. Molecular mechanism of host-endophytic fungi interaction

**Figure 1:**
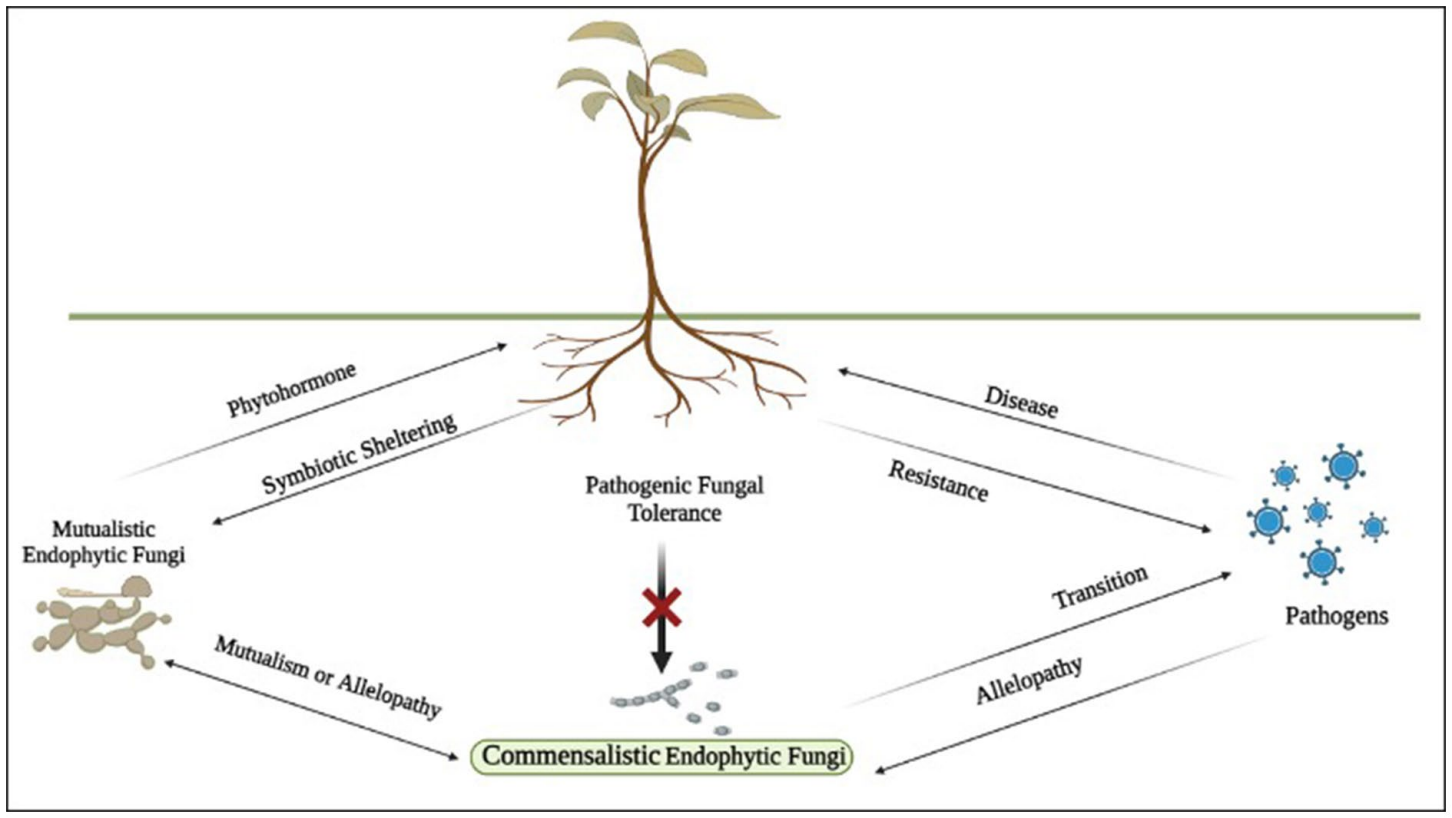
Schematic model of plant-fungi interactions.

Endophytic fungi interact with the host plant via three types of interactions, that are, mutualistic, commensalistic, and pathogenic interactions (Figure 1). In mutualistic symbiosis, both host plants and EFs benefit from each other leading to evolutionary and ecological success. The EFs on colonizing the host plant tissue alters their metabolism improving plant’s tolerance to stresses such as heavy metal and drought, augmenting growth and development, nutrient acquisition. It can also protect the host plant from herbivore animals, pests, and pathogenic microorganisms whereas the host plant provides shelter and adequate amount of nutrients to the EFs for their proliferation and life cycle completion. In commensalistic or latent pathogenic relationship, EFs interact with the host plant and may or may not show any kind of beneficial effect on the plant physiology. According to various studies, EFs reside in the host plants as dormant or latent pathogen (Brown et al., 1998; Photita et al., 2004; Rodriguez and Redman, 2008; Gorzynska et al., 2018). In other words, during this stage the fungus is harmless and does not induce any symptom of the disease but as the environmental conditions become unfavorable, latent fungi becomes active and cause obvious pathogenic symptoms eventually leading to destruction of the host plant (Romero et al., 2001; Photita et al., 2004; Poveda et al., 2020b).

In the rhizosphere, plants engage in symbiotic interactions with a diverse range of microorganisms through pattern-recognition receptors (PRRs), which are cell surface proteins capable of detecting microbial- or pathogen-associated molecular patterns (MAMPs/PAMPs) produced by the interacting microbe. These PRRs are involved in triggering the first layer of plant innate immunity. Typically, during the formation of a mutualistic symbiotic relationship, the signaling pathways that hinder the expansion of endophytic proliferation, such as miRNA-mediated pathways involved in plant defence mechanisms, are suppressed (Plett and Martin, 2018). This extracellular recognition via MAMPs/PAMPs or damage-associated molecular patterns (DAMPs) has been identified to lead to first layer of innate immunity via triggered defences, and is coined as pattern-triggered immunity (PTI) (Tang et al., 2017; Saijo et al., 2018). Another PTI can be activated upon cellular disintegration through degradation of the plant cell wall compounds such as oligonucleotides, cellodextrins, and the compounds released under stress conditions (cutin monomers and small peptides), which in turn generates endogenous signals termed DAMPs that are also detected by PRRs. Upon infection of the host plant by a microorganism, the botrytis-induced kinase1 (BIK1) effector kinase of the pattern recognition receptors (PRRs) complex becomes activated. This activation leads to an increase in the cytosolic calcium (Ca^2+^) level mediated by cyclic nucleotide-gated channels (CNGCs). This elevation of Ca^2+^ is recognized as a crucial signal for triggering the activation of pathogen-associated molecular pattern (PAMP) signals involved in plant immunity during pathogen-associated molecular pattern-triggered immunity (PTI) (Tian et al., 2019). Establishment of successful infection by the pathogen by overcoming PTI via effector-triggered susceptibility (ETS) leads to activation of plants’ second immune system termed effector-triggered immunity (ETI) which is an amplified and robust defense system of the plant. Both Pathogen-Triggered Immunity (PTI) and Effector-Triggered Immunity (ETI) are plant defence mechanisms that respond to microbe invasion. These mechanisms alter the ionic balance, leading to increased cytosolic Ca^2+^ and apoplastic reactive oxygen species (ROS) levels. Additionally, they activate the mitogen-activated protein kinase (MAPK) pathway and cause the accumulation of nitric oxide (NO). This cascade results in the production and release of phytohormones such as ethylene (ET), jasmonic acid (JA), and salicylic acid (SA). Furthermore, it induces stomata closure and callose deposition, and initiates transcriptional and metabolic reprogramming to facilitate the plant’s defense response (Nguyen et al., 2021).

**Figure 2:**
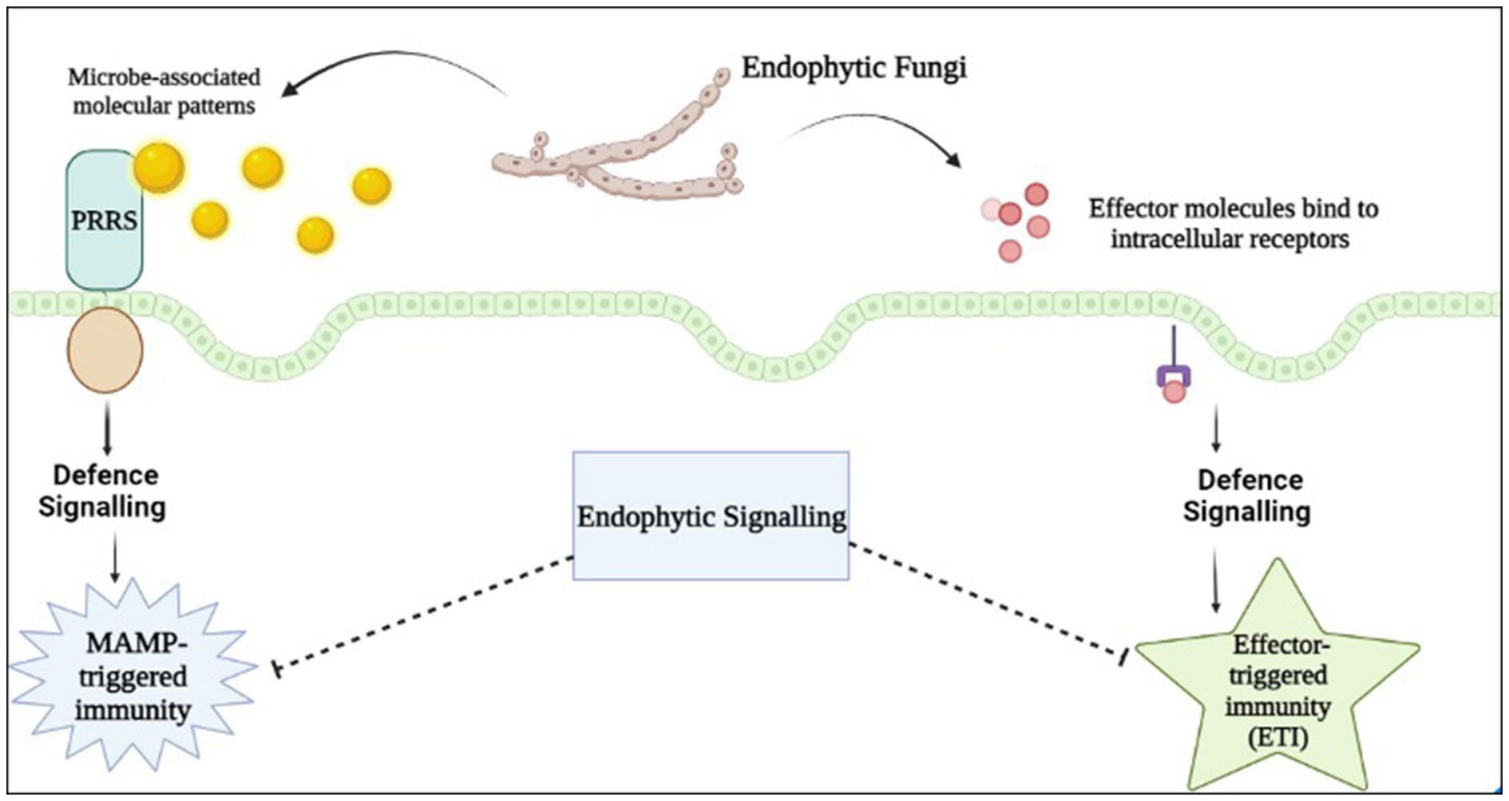
Schematic of plant immune system recognizes fungal signal molecules, resulting in two distinct defence mechanisms.

In the context of fungal endophytes, many plants have chitin-specific receptors (PR-3) on their surface that recognize the chitin oligomers found on the fungal cell wall, resulting in the activation of the plant’s defence mechanism (Sanchez-Vallet et al., 2015). Besides these, plant pattern-triggered immune signaling, such as MIN7 and CAD1, are the important components in controlling the level and nurturing the endophytes present in the nearby soil environment so that host can survive easily in the microorganism rich environment (Chen et al., 2020). In addition to various pathways, plants utilize extracellular vesicles (EVs) to facilitate the transport of signaling lipids, proteins, RNA, and metabolites between cells. Research indicates that these EVs play a role in plant stress response by exhibiting antipathogenic activity. During stress, plant cells secrete EVs containing host-derived small interfering RNAs and microRNAs that are capable of silencing fungal genes and stress responses. This evidence suggests that plant EVs may mediate trans kingdom RNA interference (Rutter and Innes, 2018). Alternately, when fungal endophytes invades the host plant, they start producing chitin deacetylases, that leads to deacetylation of the chitosan oligomers, and these oligomers are not detected by the host plant chitin specific receptors (Cord-Landwehr et al., 2016). The formation of biofilms in endophytic bacteria aids in their ability to adhere to host plant tissues and facilitate communication among themselves, thereby enabling them to evade the host plant’s defence system. Endophytic microorganisms have evolved mechanisms to evade the oxidative stress generated by the plant host in response to pathogenic invasion. The plant’s defence system includes the production of reactive oxygen species (ROS) such as superoxide (O_2_-), hydrogen peroxide (H_2_O_2_), and hydroxyl radicals (OH), which are toxic to invading pathogens. To counteract this, endophytes have developed an array of antioxidative enzymes, such as superoxide dismutases (SODs), catalases (CatAs), peroxidases (PODs), alkyl hydroperoxide reductases (AhpCs), and glutathione-S-transferases (GSTs). These enzymes play a crucial role in the elimination of ROS and the protection of the endophyte from oxidative damage, allowing it to establish and maintain a symbiotic relationship with the host plant (Zeidler et al., 2004). Endophytic microbes possess genes that encode for molecules known as Microbe-Associated Molecular Patterns (MAMPs) inhibitors. MAMPs are recognized by plants and trigger their immune response (Figure 2).

However, these inhibitors repress the MAMP-triggered immune response of the host plant. Endophytic communities can influence several physiological processes of the host plant, including the activation of silent gene clusters, leading to the synthesis of novel secondary metabolites. These secondary metabolites, which are also produced by the endophytes, play a crucial role in preventing the invasion of pathogenic microorganisms in the host plant, thereby preventing infections. The plant phenotype mostly depend upon the genetic arrangement within itself alongwith the biotic and abiotic stress and microbiome activity. The endophytic fungi stimulates the plant immune system through epigenetic events and hence, induce enhanced production of SMs leading to physiological and phenotypic change (Alam et al., 2021). Clustering of genes responsible for secondary metabolite production help in conserving the genetic arrangement. The encoding genes for these secondary metabolites are regulated by various factors such as horizontal gene transfer (HGT), transcription factors, the presence of effector molecules, and gene clustering.

#### 2.1.1. Gene clustering and transcription factors

Gene clustering can be defined as the phenomenon where the genes responsible for coding the biosynthesis of certain secondary metabolites tend to cluster together in close proximity to the telomeric or dynamic regions of the fungal chromosome. These clusters of genes can vary in two significant ways. Firstly, different clusters of genes can work together, resulting in the synthesis of highly complex secondary metabolites. Secondly, in some cases, different pathways for secondary metabolite production have gene clusters located adjacent to each other in the fungal genome (Chiang et al., 2016; Henke et al., 2016; Nützmann et al., 2018). Nevertheless, because they are involved in gene expression control, the mechanism of coding of gene clusters of secondary metabolites in unstable DNA regions remains unexplained (Osbourn, 2010a). The spatial arrangement of clustered genes is believed to be a fundamental necessity for the biosynthesis of bioactive products that are linked to a pathway. This is due to the fact that genes involved in a pathway are generally maintained in close proximity through genomic rearrangements (Deepika et al., 2016). The resulting genetic structure may allow favorable allele combinations to co-inherit at these multigene loci (Chu et al., 2011; Field et al., 2011). This may also regulate chromatin synchronization by rearranging the chromatins (Field and Osbourn, 2008; Osbourn and Field, 2009; Osbourn, 2010b).

Essentially correlated genes interspersed in higher plant genomes can form transcriptional clusters via the helix–loop–helix domain. In this, the formation of DNA loops results in the co-localization of cis-regulatory elements, leading to increased local concentrations of transcription factors at the corresponding gene’s transcription initiation sites, thereby initiating transcription. Any perturbations in the transcriptional regulation of clustered genes could result in the loss of their protein products and the accumulation of potentially harmful intermediates within the associated biochemical pathways. Many researchers showed that chromatin-level gene control is critical for the expression of secondary metabolic gene clusters (Osbourn, 2010a).

In the synthesis of secondary metabolites, clustered genes are regulated by two distinct groups of transcription factors, namely narrow domain transcription factors (NDTFs) and broad domain transcription factors (BDTFs). NDTFs exert their regulatory influence on the clustered genes directly, while BDTFs can act on the clustered genes at different gene locations than those of the NDTFs. This fact is exemplified by the renowned non-ribosomal peptide synthetase (NRPS) AflR, which is classified as a Zn_2_Cys_6_ transcription factor. AflR modulates the expression of the aflatoxin and sterigmatocystin gene clusters through its binding to the palindromic sequence 5′-TCG(N5)CGA-3′, an 11-bp motif that is present in the promoter regions of coding genes in selected *Aspergillus* species (Slot and Rokas, 2011). It also regulates three additional genes that are not associated with the aflatoxin metabolite gene cluster (Woloshuk et al., 1994; Yu et al., 1996; Cánovas et al., 2017). BDTFs, which are transcription factors that regulate the expression of multiple genes, function as high-level regulatory systems that respond to external stimuli that are not directly related to secondary biochemical gene clusters. Recent studies have revealed that ethylene-forming (EF) signals specifically disrupt transcription factors that are targeted by ethylene, indicating that these signals can influence gene expression in a variety of biological processes (Camehl et al., 2010). Thus secondary metabolites are synthesized by an organism through the interplay of developmental competence and environmental factors. These factors include, but are not limited to, nutrient supply, artificial light, pH, injury, infection, and developmental changes that occur during different stages of the host’s life cycle. It is widely accepted that a combination of these factors is essential for the efficient biosynthesis of secondary metabolites (Bayram and Braus, 2012; Xie et al., 2019). BDTFs are essential for transmitting environmental signals to the genome. They create and govern signaling transmission from environmental inputs to cellular responses during the creation of distinct SMs.

Several studies have investigated the molecular mechanisms underlying the relationship between Nucleosome-Dependent Transcription Factors (NDTFs) and Chromatin-Dependent Transcription Factors (BDTFs). These investigations aim to elucidate how BDTFs, or global transcription factors, perceive developmental or environmental cues, and subsequently transmit these signals to NDTFs through various biochemical pathways, such as chromatin and histone modifications, or through specific biochemical cascade reactions like methylation, phosphorylation, and acetylation. These responses are crucial in activating silent genes associated with specific secondary metabolites (SMs), which are required for specific cell metabolisms, growth stages, or environmental conditions. Furthermore, in addition to the plant’s genetic makeup, plant phenotypes are also influenced by the activity of the microbiome and environmental factors. This can lead to genetic changes in the appearance of endophytic fungi associated with the plant (Jia et al., 2016). It has been seen that the presence of EFs helps to improve host plant resistance to biotic and abiotic stresses (Rodriguez et al., 2008; Xie et al., 2019; Yan et al., 2019). While the precise processes are unknown, evidence shows that the presence of EFs alters plant genetic expression patterns (Mejia et al., 2014; Xie et al., 2019). Epigenetic interactions between the host and endophyte can modulate the host’s genomic expression. Endophyte-mediated alterations in DNA methylation and demethylation can enhance the host’s defence mechanisms in epigenetic processes (Geng et al., 2019). EFs strengthen plant immune systems and increase the quantity of SMs, which may cause physiological changes in infected host plants (Xie et al., 2019). Bailey et al. (2006) reported that exposure to EFs leads to changes in gene expression patterns in both the endophytic microorganisms and host organisms. These changes occur due to a complex system of genetic interactions between the EFs and hosts, resulting in altered genomic expressions during the interference process.

#### 2.1.2. Horizontal gene transfer

Horizontal gene transfer (HGT) is the process by which genetic elements are transferred between isolated lineages without sexual reproduction. This unique evolutionary mechanism is considered a novel adaptive trait of effector proteins, enabling them to invade, degrade, and manipulate host organisms (Soanes and Richards, 2014). HGT can facilitate the acquisition of a new and complete metabolic pathway by transferring the metabolic cluster of an organism to another (Slot and Rokas, 2011). The mechanism of HGT remains to be debated as to how the expression is initiated in the recipient organism. Slot and Rokas (2011) reported the successful functional transfer of a gene cluster responsible for the biosynthesis of sterigmatocystin, a toxic secondary metabolite, from *Aspergillus* species to *Podospora anserina*. The presence of certain metabolites, such as djalonensone in various *Alternaria* fungi, and aureonitol in *Chaetomium* sp. and extracts of *Helichrysum aureonitens*, which originated from horizontal gene transfer or genetic recombination during the coevolution of hosts and endophytes, provide evidence for the development of genetic regulatory mechanisms of secondary metabolite biosynthesis (Aly et al., 2010; Kozyrovska, 2013).

Through the application of the aforementioned techniques are applied by human beings for secondary metabolite production, fungal endophytes are capable of synthesizing a diverse range of secondary metabolites, including alkaloids, terpenoids, polyketides, phenylpropanoids, lignins, flavonoids, saponins, phenols and phenolic acids, aliphatic and chlorinated metabolites, peptides, and steroids. These secondary metabolites play a critical role in enabling the host plant to combat a range of stress conditions, such as drought, salinity, nutrient deficiency, metal toxicity, and biotic stress caused by pathogenic microorganisms present in the plant’s immediate environment. The plant’s capacity to endure stressors protects it from harm and prevents destruction. Elicitors or elicitor factors can even induce the synthesis of secondary metabolites with potential applications in various industries, including but not limited to pharmaceuticals, agriculture, and cosmetics. These secondary metabolites may have commercially valuable properties such as antibiotic, anti-carcinogenic, cytotoxic, insecticidal, and allelopathic activities. Some specific examples of important secondary metabolites synthesized by elicitor factors are outlined in the table below (Table 2).

**Table 2:** Secondary metabolites produced by endophytic fungus with their respective structure and functions.

Secondary Metabolites	Endophytic Fungi	Function	Reference
Terpenoids	*Pestalotiopsis microspora*, *Penicillium brevicompactum, Aspergillus terreus, Fusarium oxysporum, Colletotrichum gloeosporioides*	Participates in moderating cross-talk between endophytic fungi-host plants and also intervenes in the endophytic fungi-microbiome interaction. Act as signaling molecules between fungal-bacterial interaction and at times also aids in plant-growth promotion. These have different bioactive attributes like anti-microbial, anti-viral, anti-parasitic, antioxidant, anti-inflammatory, anticancer, herbicidial, and hypoglycemic properties.	de la Porte et al. (2020), Schulz-Bohm et al. (2017), Ditengou et al. (2015), Kaddes et al. (2019), and Ancheeva et al. (2020)
Steroids	*Alternaria alternata, Aspergillus fumigatus, Colletotrichum gloeosporioides, Xylaria sp. Neosartorya sp., Nodulisporium sp.*	Lipid-based biologically active compound having a range of anti-inflammatory, anti-cancer, anti-parasitic properties. Few examples include ergosterol, stigmasterol, campesterol, 22-hydroxy-cholesterol, brassicasterol, aspergilolide	Alam et al. (2021), Zhang et al. (2019), Nowak et al. (2016), and Yang et al. (2015)
Xanthones	*Aspergillus fumigatus, Phomopsis sp., Pestalotiopsis sp., Diaporthe sp., Colletotrichum gloeosporioides, Exserohilum rostratum*	Polysubstituted polyketide derivatives with a wide spectrum of pharmacological activities. Xanthones and its derivatives have potential, phytotoxic, cytotoxic, anti-protozoal, antibacterial properties. Also few xanthones like vertixanthone, danthron, globosuxanthone have shown antifungal activity against phytopathogenic fungi.	Ming et al. (2022), Pina et al. (2021), Miao et al. (2020), and Kaddes et al. (2019)
Quinones	*Alternaria sp., Fusarium sp., Xylaria sp., Phoma sp., Pestalotiopsis sp., Aspergillus sp., Talaromyces assiutensis*	These have a wide spectrum of medicinal properties. Phomol and phomenone are types of quinone known for their antibacterial properties. Other derivatives Xylarione, Pestaloside, Fusaric Acid are reported to have anti-tumor activities and hence are being studied for being potential anti-cancer drugs. These also possess antioxidant and antiviral attributes.	Lou et al. (2013), Christiansen et al. (2021), Mishra R. C. et al. (2021), and Ancheeva et al. (2020)
Phenols	*Colletotrichum gloeosporioides, Taxomyces andreanae, Phomopsis sp., Curvularia lunata, Cryptosporiopsis sp., Aspergillus flavus, Pestalotiopsis mangiferae, Vernonia amygdalina*	There are a variety of properties of different phenolic compounds extracted from endophytic fungi.Taxol, for example, has found extensive use in chemotherapy for its anti-cancer property. Similarly, resveratrol, gallic acid, curcumin, catechin has been found to exhibit significant anti-inflammatory, anti-oxidant and anti-microbial attributes.	Ancheeva et al. (2020), Praptiwi et al. (2020), Yadav et al. (2014), and Subban et al. (2013)
Mycorrhizin	*Plectophomella sp., Pezicula sp.*	These secondary metabolites exhibit significant cytotoxic, nematicidal, antibacterial antifungal properties. Also having such distinct antimicrobial activities reflects potential usage in food industries.	Adeleke and Babalola (2021), Preethi et al. (2021), McMullin et al. (2017), Hussain et al. (2014), and Schulz et al. (2015)
Furandiones	*Aspergillus fumigatus, Aspergillus terreus, Fusarium solani, Fusarium graminearum, Phoma sp.*	These secondary metabolites are characterized by the presence of a furan ring. It is suggested these metabolites have a role in host-fungus interaction by protecting the host plant against phytopathogenic invasion. Furandione like beauvericin, phomodine, terrein, camptothecin, fumitremorgin C, have antifungal, antiviral, antibacterial properties.	Wang et al. (2022), Pavithra et al. (2020), Garcia et al. (2012), Ding et al. (2019), and Rajashekar et al. (2016)
Isocoumarin	*Xylaria grammica, Xylaria mali, Xylaria cubensis, Ampelomyces sp., A. truncatum, Xylariaceae sp., Geotrichum sp., Aspergillus banksianus*	These are bioactive molecules having a vast arena of antimicrobial, algicidal, antimycobacterial, antiplasmodial, antiviral, antimalarial activities. Also these have plant growth regulatory functions along with protein kinase inhibitory acetylcholinesterase, and glucosidase activity. Different derivatives of isocoumarins have also been reported to have significant anti-inflammatory, anti-oxidant, and cytotoxic properties.	Lv et al. (2023), Cheng et al. (2020), and Noor et al. (2020)

## 3. Endophytic fungi as source of terpenoid production

Endophytic fungi are capable of producing a diverse range of bioactive compounds that exhibit a wide array of biological activities including, but not limited to, insecticidal, antioxidant, antifungal, antiviral, antibacterial, and cytotoxic properties. These compounds belong to various chemical classes such as terpenoids, phenols, alkaloids, polyketides, quinones, steroids, enzymes, and peptides. The secretion of these bioactive compounds by endophytic fungi is known to host plant defence response, enabling it to better cope with both biotic and abiotic stressors. Methods to enhance the potential of endophytic fungi for producing these secondary metabolites include the activation of silent biosynthetic pathways, epigenetic modifications, and other techniques. Among these secondary metabolites, terpenoids have emerged as a particularly important class of molecules that have significant applications in human health and agriculture. Thus, endophytic fungi represent a promising novel source for the bioproduction of these valuable terpenoid compounds. The isoprene units are typically obtained through two pathways, the mevalonic acid pathway (MVA) and the methylerythritol phosphate pathway (MEP). Based on the number of carbon atoms in their skeletal structure [(C_5_)_n_], terpenoids can be classified into various subgroups, including hemi terpenoids (C_5_), monoterpenoids (C_10_), sesquiterpenoids (C_15_), diterpenoids (C_20_), sesterterpenoids (C_25_), triterpenoids (C_30_), and tetraterpenoids (C_40_) (Gershenzon and Dudareva, 2007).

The building blocks of terpenoids, which are a diverse group of natural products, are isopentenyl pyrophosphate and dimethylallyl pyrophosphate. These are synthesized through the mevalonate (MVA) and the methylerythritol phosphate (MEP) pathways, and are interconvertible by the action of isopentenyl pyrophosphate isomerase. Subsequently, prenyltransferases convert these shorter chain precursors to longer chain terpenoid skeletons, such as geranyl diphosphate, farnesyl diphosphate and geranylgeranyl diphosphate, which serve as the C10, C15, and C20 backbones of monoterpenoids, sesquiterpenoids and triterpenoids, diterpenoids and tetraterpenoids, respectively. Further structural diversification of terpenoids can be achieved by the introduction of functional groups, such as glycosyl, hydroxyl, ketone, carbonyl, and aldehyde, or by rearrangement of the carbon skeleton. These modifications can lead to the expression of various bioactivities associated with terpenoids (Sun et al., 2019). Numerous plant species are recognized for their capacity to biosynthesize terpenoids, including citral, menthol, camphor, salvinorin A (in the case of the plant *Salvia divinorum*), ginkgolide, and bilobalide (in the case of the plant *Ginkgo biloba*), and cannabinoids (in the case of the cannabis plant). Terpenoids are also commonly synthesized by tea, thyme, Spanish sage, and citrus fruits (such as lemon, orange, and mandarin). These terpenoids exhibit diverse functions in the food and cosmetic industries, as well as various pharmaceutical and nutritional applications. Furthermore, terpenoids have demonstrated good antiviral activity, and have therefore been investigated for their potential clinical use in the treatment of COVID-19. Due to these activities, there is an increase in demand of terpenoids which possess stress on the terpenoids producing plant species. In order to fulfil the increasing demand of terpenoids, other sources which are environmentally friendly and commercially fulfilling need to be discovered. The endophytes have the potential of synthesizing these terpenoids which have gained the attention for using the endophytes for alternate source of terpenoid-producing strains or terpenoid synthetic genes (Chen et al., 2021).

Endophytic fungi and its host plant undergo various complex interactions which enhance the plants growth and nutrition, also helps the plant in combating various biotic and abiotic stress conditions. Endophytes are known to exhibit a high degree of variability that is dependent on several factors such as the genotype of the host plant, growth stage, physiological state, tissue type, soil environment, and various agricultural practices (Gupta et al., 2020). These microorganisms have been shown to not only produce terpenoids themselves, but also stimulate their production in host plants through mechanisms such as horizontal gene transfer, the heterologous expression of terpenoid biosynthetic genes sourced from endophytes, biotransformation of terpenoids, and various signaling pathways that include elicitor recognition, signal transduction, integration with transcription factors, and gene activation (Zhai et al., 2017). Numerous scientific methodologies have been created to advance terpenoid biosynthesis research. These include metabolic engineering and synthetic biology, system biology approaches, modern metagenomic sequencing methods, and the *de novo* assembly of microbial genomes from metagenome data. These techniques enable the expression of the terpenoid biosynthetic pathway in industrial microbes, increase the potential gene pool involved in terpenoid biosynthesis from various environments, and offer effective strategies for discovering novel microbes and genes related to terpenoid biosynthesis in the endosphere and rhizosphere microbiomes, regardless of their culturability (Carrión et al., 2019; Belcher et al., 2020). Huperzine A (HupA) is a naturally occurring sesquiterpene alkaloid that is synthesized by members of the Huperziaceae family, which includes species such as *Huperzia serrata*. HupA has been found to possess potent anti-acetylcholinesterase activity, making it an effective treatment option for Alzheimer’s disease (AD). Furthermore, HupA’s ability to effectively inhibit acetylcholinesterase has been associated with minimal side effects, highlighting its potential as a safe and effective treatment option for AD (Zhao et al., 2013). The enzymes lysine decarboxylase and copper amine oxidase catalyze the first two steps of HupA biosynthetic pathway in which L-lysine is converted to 5-aminopentanal which is the precursor of HupA. Researchers successfully isolated two strains of endophytic fungi, EFs *Shiraia* sp. Slf14 (Zhu et al., 2010) and *Cladosporium cladosporioides* LF70 (Zhang et al., 2019), from the leaves of *Huperzia serrata*. These strains were found to produce Huperzine A (HupA) and were able to yield 142.6 μg/g and 39.61 μg/g dry mycelium, respectively. On sequencing the whole genome of *Shiraia* sp. Slf14, HupA biosynthetic gene cluster was identified, which is then expressed into *Escherichia coli*, which showed that genetically modified *E. coli* strain was able to convert cadaverine to 5-aminopentanal (Yang et al., 2016). Afterwards, some other strains of EFs were isolated, such as *Colletotrichum gloeosporioides* ES026, which were then sequenced and expressed for its yield of 5-aminopentanal which is a precursor of HupA.

Traditionally utilized medicinal plants *Nothapodytes nimmoniana* and *Campotheca acuminata* contain campothecin which belongs to the class of monoterpene indole alkaloids and functions as potent topoisomerase inhibitor in DNA replication. Reports from various studies suggest that *Entrophospora infrequens* and *Neurospora crassa* isolated from *N. nommoniana* produce campothecin (Puri et al., 2005; Rehman et al., 2008). Kusari et al. (2009) suggested that *Fusarium solani* isolated from the barks of *C. acuminata* are potential source of campothecin production. The endophytic strain of *F. solani* utilizes enzymes encoded by its gene in presence of strictosidine synthase, one of the majorly important plant enzyme for the campothecin biosynthesis. Another report by Yan et al. (2019) suggested that ginsenosides Rh2 and Rg3 are produced by strain PDA-2 which is closely related to *Agrobacterium rhizogenes* which was isolated from *Panax ginseng*. The ginsenosides Rh2 and Rg3 have major roles in inhibiting tumor cell proliferation and induce apoptosis. In a study by Palem et al. (2015), authors have suggested biosynthesis of vincristine and vinblastine by endophytic fungus *Talaromyces radicus* isolated from various tissues of *Catharanthus roseus*. The study screened 22 endophytic fungi for presence of gene encoding tryptophan decarboxylase which could only be amplified in *T. radicus*. The gene tryptophan decarboxylase is one of the most important enzyme terpenoid indole alkaloid biosynthetic pathway. Maximum yield of 670 μg/L and 70 μg/L of vincristine and vinblastine from modified M2 medium and PDB medium was obtained, respectively. Parthasarathy et al. (2020) recorded the vinblastine production *Curvularia verruculosa* isolated from leaves of *C. roseus*. Maximum yield of 182 μg/L of vinblastine was obtained from endophytic fungi *C. verruculosa*.

## 4. Biotic stress regulation and terpenoids

Stress can be defined as any substance, microorganism or unfavorable condition which directly or indirectly affects the plant’s growth and development, metabolism or nutrition. Stress on vegetation depends on factors such as, temperature, mineral deficiency, long rainy periods, desiccation problems, insects and pathogens, herbicides and pesticides, pollutants, climatic conditions like drought or floods, increased UV radiations and so on. Based on the LICHTENTHALER model of stress in plants (Lichtenthaler, 1998), stress can be divided into several distinct phases. The first phase is the response phase, which is characterized by an alarm reaction and marks the beginning of stress. This is followed by the restitution phase, where the plant enters a stage of resistance and continues to experience stress. The third phase is the terminal phase, where the plant enters a stage of exhaustion after long-term stress. Finally, the regeneration phase allows the plant to recover and rejuvenate after experiencing stress. These phases of stress describe in detail the changes that occur in the plant during the unfavorable or stress conditions. Initially, there is decline in physiological functions which leads to decrease in metabolic activities and decline in growth, development and vitality of the plant. In response, the plant’s defence system gets activated leading to hardening of the plant by increasing the plant’s resistance against stressors by reaching plant resistance maximum. If the stressors are removed before senescence the plant survives via regeneration but if the stressors are not being removed then the plant leads to cell death which ultimately causes plant death (Lichtenthaler, 1998). Plant stressors can be divided into three types, that is, natural or abiotic stressors such as high light, heat, drought, mineral deficiency, low temperature, chilling, wounding, UV-A and UV-B, biotic stressors such as insects, pathogens, elicitors, bacteria, fungi and virus, anthropogenic stressors such as, herbicides, air pollutants, peroxyacyl nitrates, free radicals, acid rain, acid fog and heavy metal load. Plants sense these stressors and act accordingly via activation of signal transduction pathways after signal perception further leading to gene expression or metabolic responses in the plant. Under stress conditions, plants synthesize bioactive molecules such as phytohormones and secondary metabolites which not only have the tendency to overcome the stress conditions but also have antifungal, anticarcinogenic and various other medicinal properties which can be utilized to treat various diseases. EFs have the capability of producing the secondary metabolites (terpenoids, alkaloids, phenols, quinones) that would help in fighting the biotic and abiotic stress in plants as well as can be utilized in pharmaceutical industry due to its medicinal properties (Gershenzon and Dudareva, 2007; Christiansen et al., 2021).

When endophytic fungi interact with their host plant, the plant’s defence system is activated to counteract the presence of the endophytic fungi which are perceived as potential pathogens. However, endophytic fungi are capable of evading these defence mechanisms and colonizing the host plant. Subsequently, endophytic fungi produce bioactive compounds, such as terpenoids, phenols, alkaloids, steroids, quinones, poly-ketones, and peptides, which exhibit inhibitory effects on the growth of pathogens and herbivores invading the host plant in the presence of endophytic fungi (Lu et al., 2021). This phenomenon provides a protective mechanism to the host plant from pathogenic microorganisms. Various terpenoids are synthesized by endophytes which will help host plants to combat biotic and abiotic stresses. Examples of these terpenoids include indole diterpenoids, synthesized in endophytic infected grasses, are neurotoxins, responsible for intoxication, helpful in protecting the grass from cattle grazing (Hardoim et al., 2015) multi-cyclic indolosesquiterpene synthesized by *Streptomyces* sp. HKI0595 in *Kandelia candel* has antibacterial activity (Maier et al., 2011), cadinane sesquiterpene derivatives synthesized by *Phomopsis cassia* in *Cassia spectabilis* has antifungal activity against phytopatogenic fungi *Cladosporium cladosporioids* and *C. sphaerospermum. Persicaria minor* also known as kesum is herbaceous plant widely found in south-east Asia and is mainly important for flavonoid and terpenoid synthesis. Study by Samad et al. (2019) reported that six miRNAs of *Persicaria minor* post-transcriptionally regulated the terpenoid biosynthesis induced by *Fusarium oxysporum*.

## 5. Multi omics technology to uncover secondary metabolites from endophytes

A large diversity of microorganisms is present in the soil that interacts with the plants growing in that soil. These interactions are known as plant-microbe interaction. These interactions play a very important role in making a sustainable balanced ecosystem. Plants synthesize various organic and inorganic nutrients which makes the soil nutrient enriched that is very beneficial for the microbial consortia growing in that soil. Plants house endophytes which are one of the examples of plant-microbe interaction. Such interactions between plants and various microbes such as bacteria and fungi are beneficial and attract the interest of various researchers due to their potential for combatting biotic and abiotic stresses along with its application in agriculture. Throughout their life cycle, plants engage in the synthesis of two distinct categories of metabolites: primary and secondary metabolites. Primary metabolites, which are essential for the plant’s growth, development, and nourishment, remain constant across all plant species. In contrast, secondary metabolites function as signaling molecules that play a crucial role in signal transduction. These metabolites are typically secreted by plants in response to herbivore, pathogen, or environmental stress but in low quantities. However, research suggests that endophytes, particularly EFs, have the capability to produce these secondary metabolites in large quantities when compared to plants. There are several researchers working for the discovery of these secondary metabolites as these are potential source for depleting the use of chemical pesticides, insecticides or herbicides and these discoveries are categorized as: (a) metabolites that are unknown, (b) metabolites with known function but unknown structure, (c) metabolites with unknown function but known structures (Luo et al., 2022).

Researchers face several difficulties in the discovery of these secondary metabolites which include (1) metabolites are secreted by the plants in very low concentrations, (2) the metabolites that are being secreted and synthesized are being actively metabolized as well, (3) they have diverse physical and chemical properties which require specific assays to determine their functions and structures, (4) these metabolites are secreted at specific stages of the plant’s life which makes it difficult for scientists to discover them (Luo et al., 2022). To surmount the hurdles in the study of plant secondary metabolites, it is imperative to adopt interdisciplinary methods. A multi-omics approach, for instance, has been instrumental in the identification of these metabolites via the methodical comparative analysis of extensive datasets by researchers. In a broader aspect, “omics” are the scientific fields that are related to measuring biological molecules in high throughput methods. This includes various biological fields such as genomics, proteomics, metagenomics, metabolomics, phenomics, epigenomics, transcriptomics and so on. When all these fields are studied in an integrated manner for discovery of some biological molecule, then it is called a multi-omics approach. This review utilizes a multi-omics approach to investigate a diverse range of secondary metabolites synthesized by endophytes. By employing one or multiple omics approaches, a scientific experiment can be designed to align with the function and structure of the metabolite. Each omics approach has its unique characteristics and can complement the limitations of the other omics approach, thereby providing a comprehensive understanding of the endophytic secondary metabolite’s synthesis and its potential applications.

Genomics is the field of biology that deals with the study of the whole genome (including structure, function, evolution, mapping and editing of genome) of the organism. Genome is the whole sequence of the DNA set of the organism. The fundamental genetic material, DNA, contains essential information about the regulation of gene expression, including promoter regions, untranslated regulatory regions, and splicing sites, as well as the protein-coding sequence that determines the function of a given gene within an organism (Chu et al., 2020). The field of genomics is driven by advanced technologies such as high-throughput DNA sequencing techniques, such as Illumina HiSeq, PacBio, and Nanopore sequencing, as well as single nucleotide polymorphism (SNP) chips that enable the identification and analysis of genetic variations among individuals. The whole genome sequencing analysis would help us understand that which genes are responsible for growth and development, defence system, nutrient acquisition, production of secondary metabolites, and various other processes of the organism which we are studying. This approach of whole genome sequencing would help in determining the genes that are directly or indirectly influencing the various bioactivities or metabolic activities of the endophyte or plant which is synthesizing the secondary metabolites.

In the context of the interactions between endophytes and plants, various biotic and abiotic stresses may occur during their interactions leading to the production of secondary metabolites as a defence mechanism. Genomics approaches utilizing high-throughput sequencing techniques offer a powerful tool for identifying the specific genes responsible for the activation of signaling pathways leading to the production of secondary metabolites (Alam et al., 2021). Additionally, such approaches can be used to investigate plant growth promotion, endophytic secretory systems, surface attachment and insertion elements, transport systems, and other related metabolic mechanisms. This detailed knowledge about the genes and its functions would provide us with better understanding about the ecology and evolution of the endophyte and we could accordingly extract the gene responsible for secondary metabolite production and amplify it using PCR for its further utilization. Compounds like alkaloids, flavonoids, minerals, polyphenols and vitamins are mostly synthesized by endophytes have positive influence on adjusting in adverse conditions and hence, promoting plant health (Balestrini et al., 2021). These molecules/compounds can be detected and analyzed employing techniques such as gas chromatography–mass spectroscopy (GC–MS), Fourier transform infrared (FT-IR), nuclear magnetic resonance (NMR) spectroscopy, metabolite fingerprinting, time-of-flight mass spectrometry (TOF-MS), Orbitrap Mass Spectrometer (Orbitrap-MS), flash chromatography. The signaling pathway initiation and colonization factors due to host cell and endophytic interactions can be evaluated on the basis of proteomics studies. However, there are new omics tools available for deciphering the fungal genome and the metabolites produced by them through epigenomics, ionomics, fluxomics, lipidomics, nutrigenomics, toxicogenomics. By integrating these various techniques, the secondary metabolite production can be verified and analyzed at every level (Table 3; Shankar and Sharma, 2022). The host endophyte interaction from recognition phase to stress resistance development can be thoroughly studied via transcriptomic analysis.

**Table 3:** Selected studies on secondary metabolites based on omics technology.

Host Plant	Endophyte	Technique	Platform used for the study	Metabolites analyzed	Reference
*Arabidopsis thaliana*	*P. variotii*	Transcriptomics	-	Salicylic acid	Lu et al. (2019)
*Zea mays*	*T. virens*	Transcriptomics	NovaSeq 6,000	Salicylic acid and Jasmonic Acid	Malinich et al. (2019)
*Anoectochilus roxburghii*	*Ceratobasidium* sp. AR2	Transcriptomics; Metabolomics	Illumina HiSeq 2000; HPLC–MS/MS	Flavonoid biosynthetic genes; Flavonol glycosides	Zhang et al. (2020)
*Panax quinquefolius*	*Conexibacter* sp.	16S rRNA sequencing; Metabolomics	Illumina NovaSeq platform; LC–MS/MS	Saponin biosynthetic genes	Li et al. (2023)
*A. thaliana*	*B. megaterium* (BT 22)	Transcriptomics; Metabolomics	DNBSEQ high-throughput platform; LC–MS/MS	Auxin response genes, flavonoid biosynthetic genes	Liu et al. (2023)

During the investigation of the metabolic output of the colocalization phenomenon of prenyltransferase (Polyprenyl synt (PT)) and terpene synthase (Terpene synth C (TPS)) domains in plant genomes, the biosynthesis of sesterterpenes, a rare type of terpenoid, was discovered to occur in both plants and fungi (Huang et al., 2017, 2018). *P. indica*, has the potential of acting as plant probiotic agent, that has been revealed during its genome sequence analysis (Qiang et al., 2012). EFs of order sebacinales helps the host plant in enhancing its growth, development and stress tolerance potential, is revealed by this approach (Weiss et al., 2011). Several bioinformatic tools, such as plantiSMASH (Kautsar et al., 2017), phyto cluster (Töpfer et al., 2017), and clusterfinder (Schläpfer et al., 2017; Chavali and Rhee, 2018), have been developed to predict plant biosynthetic gene clusters (BGCs) using plant genomic sequences, protein annotations, and gene expression profiles. This facilitates the identification of plant secondary metabolites, which can have important implications in fields such as drug discovery and agriculture.

## 6. Future prospect and conclusion

The development of a sustainable agriculture is of utter importance. Microorganisms like the endophytic fungi represent an intriguing area of study in the field of plant-microbe interactions in this context. The unique ability of these fungi to inhabit and colonize host plant tissues and thereby stimulate the production of wide range of secondary metabolites with diverse biological activities, including antifungal, antibacterial, anticancer, and immunomodulatory properties are of extreme importance. These attributes enhance the plants disease resistance ability, growth and nutrient uptake abilities. Most of these secondary metabolites produced by these fungi are valuable sources of antimicrobial, antitumor, and antiviral agents, among other bioactive compounds, making them a vital component in the development of new drugs. Multi-omics technologies can further help in deciphering the physiological development of endophytes in host plant. Concomitantly, generation of more high-throughput data will provide updated information on the core areas of study, thus discovering unknown genes, metabolites, and microbial species for better harnessing of beneficial aspects from endophytes. Thus, in conclusion it can be well understood that the overall, investigation of endophytic fungi and their metabolic products has gained significant attention in recent years, and this approach holds great promise for sustainable agriculture. With the increasing demand for eco-friendly and non-toxic agricultural practices, endophytic fungi present an attractive alternative to chemical pesticides and fertilizers. Further studies on these fungi will not only provide a better understanding of their interactions with host plants but also contribute to the development of sustainable agricultural practices and the discovery of novel bioactive compounds for various applications.

## Author contributions

PJ and TM: conceptualization, editing, and supervision. TK and IC: literature review and drafting of original manuscript. AP, SP, and VK: writing and editing. All the authors have read and approved the final version of the manuscript.

## Conflict of interest

IC was employed by Metropolis Healthcare Ltd.

The remaining authors declare that the research was conducted in the absence of any commercial or financial relationships that could be construed as a potential conflict of interest.

## Publisher’s note

All claims expressed in this article are solely those of the authors and do not necessarily represent those of their affiliated organizations, or those of the publisher, the editors and the reviewers. Any product that may be evaluated in this article, or claim that may be made by its manufacturer, is not guaranteed or endorsed by the publisher.

## Figures and Tables

**Table:** 

VOCs	Volatile Organic Compounds
PRRs	Pattern-Recognition Receptors
MVA	Mevalonic Acid Pathway
MEP	Methyl Erythritol Phosphate
NGS	Next Generation Sequencing
EF	Endophytic Fungi
MAMPs/PAMPs	Microbial- or Pathogen-Associated Molecular Patterns
BIK1	Botrytis-Induced Kinase1
EVs	Extracellular Vesicle
DAMPs	Damage-Associated Molecular Patterns
CNGCs	Cyclic Nucleotide-gated Channels
PTI	Pattern-Triggered Immunity
HGT	Horizontal Gene Transfer
ETI	Effector-Triggered Immunity
NDTFs	Narrow Domain Transcription Factors
BDTFs	Broad Domain Transcription Factors
SNP	Single Nucleotide Polymorphism
BGCs	Biosynthetic Gene Clusters
AD	Alzheimer’s disease
GC–MS	Gas Chromatography–Mass Spectroscopy
FT-IR	Fourier Transform Infrared
NMR	Nuclear Magnetic Resonance Spectroscopy
TOF-MS	Time-of-Flight Mass Spectrometry
Orbitrap-MS	Orbitrap Mass Spectrometer
